# Naturalistic Stress Hormone Levels Drive Cumulative Epigenomic Changes along the Cellular Lifespan

**DOI:** 10.3390/ijms22168778

**Published:** 2021-08-16

**Authors:** Anthony S. Zannas

**Affiliations:** 1 Department of Psychiatry, University of North Carolina, Chapel Hill, NC 27514, USA; anthony_zannas@med.unc.edu; Tel.: +1-(919)-962-4918; 2 Department of Genetics, University of North Carolina, Chapel Hill, NC 27514, USA; 3 Carolina Stress Initiative, University of North Carolina School of Medicine, Chapel Hill, NC 27514, USA

**Keywords:** cortisol, DNA methylation, environmental stress, epigenetics, fibroblasts, glucocorticoids

## Abstract

Environmental stress is ubiquitous in modern societies and can exert a profound and cumulative impact on cell function and health phenotypes. This impact is thought to be in large part mediated by the action of glucocorticoid stress hormones, primarily cortisol in humans. While the underlying molecular mechanisms are unclear, epigenetics—the chemical changes that regulate genomic function without altering the genetic code—has emerged as a key link between environmental exposures and phenotypic outcomes. The present study assessed genome-wide DNA (CpG) methylation, one of the key epigenetic mechanisms, at three timepoints during prolonged (51-day) exposure of cultured human fibroblasts to naturalistic cortisol levels, which can be reached in human tissues during in vivo stress. The findings support a spatiotemporal model of profound and widespread stress hormone-driven methylomic changes that emerge at selected CpG sites, are more likely to spread to nearby located CpGs, and quantitatively accrue at open sea, glucocorticoid receptor binding, and chromatin-accessible sites. Taken together, these findings provide novel insights into how prolonged stress may impact the epigenome, with potentially important implications for stress-related phenotypes.

## 1. Introduction

Environmental stress is ubiquitous in modern societies and can exert profound impact on cell and body function [1,2]. This impact can accumulate throughout the human life and contribute to a host of disease states together responsible for 70% of all deaths [3]. Although the underlying mechanisms are unclear, epigenetics—the chemical changes that regulate genomic function without altering the genetic code—has emerged as a key link between the environment and health [4,5,6]. Studying the epigenetic sequelae of stress can thus yield fundamental insights into determinants of health and disease. In particular, DNA methylation at cytosines followed by guanine residues (CpG) is one of the most widely studied epigenetic modifications in humans and has been proposed as a key mechanism mediating the impact of stress on cell function and phenotypic outcomes [4,6,7,8,9].

While stress can result from heterogeneous physical, mental, and social stimuli, all stressors share an ability to trigger conserved neuroendocrine responses that culminate in systemic secretion of glucocorticoid stress hormones, primarily cortisol in humans [10]. Systemic glucocorticoids, in turn, can influence genomic function in essentially every human cell by activating the glucocorticoid receptor (GR), a ligand-regulated transcription factor [11]. Glucocorticoid exposure and GR activation can induce not only acute changes in gene transcription but also lasting epigenetic changes, most notably in DNA methylation [12,13,14,15,16,17,18,19]. Building on sparse existing evidence, we previously further hypothesized that prolonged or repeated exposure to stress and glucocorticoids can induce cumulative epigenetic changes at susceptible genomic sites [6,11].

To address this hypothesis and spatiotemporally characterize the cumulative epigenomic effects of prolonged stress, the present study employs a well-established line of cells (IMR-90 fibroblasts). Fibroblasts are particularly suitable in this setting, because they have a finite replicative potential (cellular lifespan) but can also be treated in culture for a long period of time (≥2 months) [20]. While the majority of prior studies have used either large concentrations of cortisol or synthetic (and much more potent) glucocorticoids [14,17,18,19,21,22,23,24,25], the present study models naturalistic stress hormone exposure using cortisol at a concentration of 100 nM, which can be reached in human tissues during in vivo stress [26,27,28,29,30]. Taken together, the findings show that prolonged exposure to naturalistic cortisol levels induces profound and widespread methylomic changes that emerge at selected CpG sites, are more likely to spread to nearby located CpGs, and quantitatively accrue at open sea, GR binding, and chromatin-accessible sites.

## 2. Results

### 2.1. Prolonged Exposure to Naturalistic Stress Hormone Levels Induces Widespread and Cumulative Methylomic Changes

**Figure 1:**
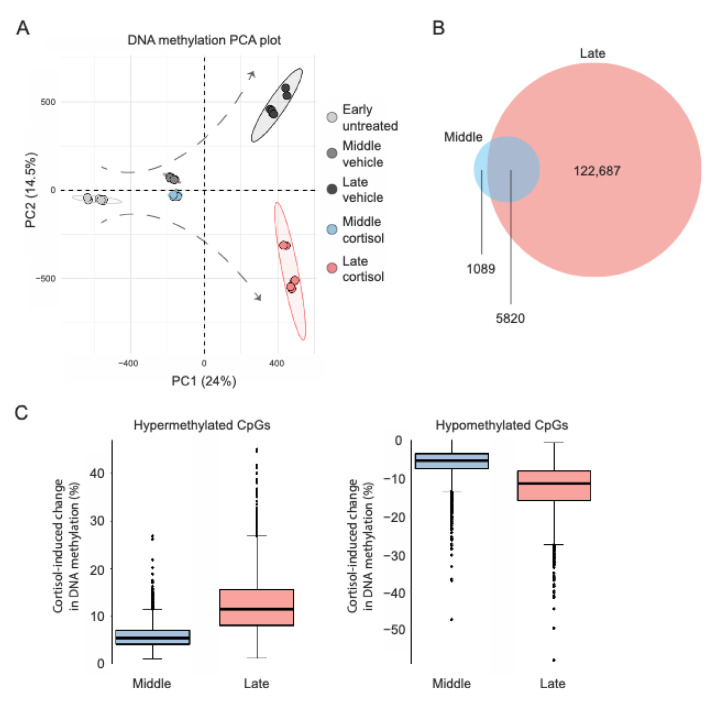
Prolonged cortisol exposure induces widespread and cumulative DNA methylation changes. (**A**). Principal component analysis (PCA) of Illumina EPIC array data showing the progression of cortisol-induced methylomic changes along the cellular lifespan. (**B**). Venn diagram depicting the number of CpG sites with significant treatment condition-duration interaction and methylation differences between cortisol and vehicle groups at middle or late passage. (**C**). Boxplots comparing the magnitude of cortisol-induced methylation changes (hyper- or hypo-methylation) at middle and late passage. Each datapoint represents a significantly changing CpG and methylation changes.

IMR-90 cells underwent prolonged exposure to either vehicle or 100 nM cortisol (as indicated below), and genome-wide DNA methylation was measured with the Illumina Infinium HumanMethylationEPIC BeadChip at three time points (cell passages): 0 days (“early”), to determine baseline methylation right before treatment onset; 24 days (“middle”), an intermediate timepoint to assess potential cumulative epigenomic effects; and 51 days (“late”), the latest time point following treatment completion. Principle component analysis (PCA) of genome-wide DNA methylation data (total 709,065 CpG sites after quality control) indicated that the effects of prolonged exposure to cortisol accumulate and shape distinct epigenomic landscapes through transition from early to middle and late passage (Figure 1A). To further characterize the spatiotemporal distribution of cortisol-induced methylomic changes, linear regression models tested if treatment condition (cortisol vs. vehicle) and duration (up to 51 days) interact to influence methylation at each EPIC array-covered CpG site. This analysis identified a total of 129,596 CpGs exhibiting statistically significant condition-duration interaction as well as significant DNA methylation differences between cortisol and vehicle groups at either middle or late passage (FDR-adjusted *p* < 0.05). Among these, significant cortisol-induced methylation changes already emerged for 6909 CpG sites at middle passage (3660 hyper- and 3249 hypo-methylated), whereas changes became significant for 128,507 CpGs at late passage (57,129 hyper- and 71,378 hypo-methylated) (Figure 1B). For the 5820 CpGs with significant methylation changes at both timepoints, the magnitude of cortisol-induced changes was much smaller for middle passage (hypermethylation mean 5.0%, SD 4.0%, max 26.9%; hypomethylation mean −5.0%, SD 5.9%, max −47.3%) as compared to late passage (hypermethylation mean 12.5%, SD 5.9%, max 45.0%; hypomethylation mean −12.7%, SD 6.3%, max −58.0%) (Figure 1C). These observations suggest that prolonged exposure to naturalistic stress hormone levels affects a gradually (and seemingly exponentially) increasing number of CpG sites, while also driving potentially cumulative methylation changes at some of the affected CpGs.

### 2.2. Stress Hormone-Driven DNA Methylation Changes Are more Likely to Emerge near already Affected CpG Sites

**Figure 2:**
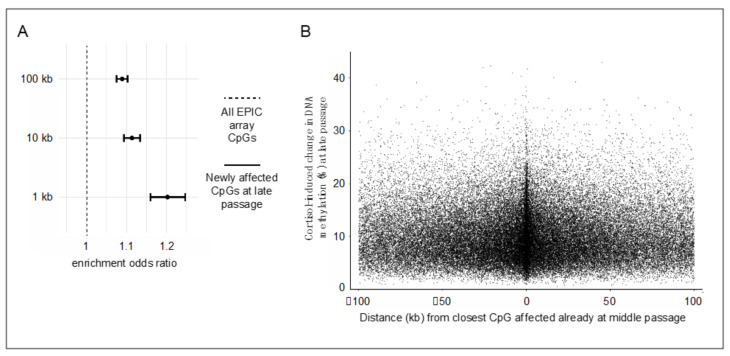
Cortisol-induced methylation changes are more likely to emerge and show greater magnitude of effect at genomic sites located near already affected sites. (**A**)**.** Enrichment analyses of cortisol-induced methylation changes newly emerging at late passage at CpG sites located within varying windows (1, 10, or 100 kb) from the closest CpG affected already at middle passage. Error bars show odds ratios and 95% confidence intervals, which were calculated with Fisher’s exact test. (**B**). Scatterplot depicting the relation between absolute cortisol-induced DNA methylation changes at newly affected CpGs at late passage and distance from the closest CpG affected already at middle passage. Each datapoint depicts a significantly changing CpG.

The observation that prolonged stress hormone exposure affects an exponentially increasing number of CpG sites warrants further examination. Because DNA methylation patterns, once established, can influence local chromatin accessibility [14,17,19,31], it can be hypothesized that the initial methylation changes induced by cortisol at susceptible CpG sites could facilitate subsequent additional changes at proximally located CpGs. To address this “epigenetic seeding and spreading hypothesis”, analyses examined the likelihood of additional cortisol-induced methylation changes to emerge at late passage (“spreading”) at CpG sites located within varying windows (1, 10, or 100 kb) from the closest CpG affected already at middle passage (“seeding”). These analyses used all EPIC array-covered CpG sites as a background (excluding the ones identified as seeding sites) and showed that epigenetic spreading is more likely to occur at CpGs located near seeding sites for all selected windows (all *p*-values < 2.2 × 10^−16^; Figure 2A). Moreover, the magnitude of methylation changes at spreading CpGs inversely correlated, albeit weakly, with the distance from the closest seeding CpG (Figure 2B; Spearman ρ = −0.08, *p* = 5.7 × 10^−7^ for the 1-kb window). These results suggest that stress hormone-driven methylation changes are more likely to emerge and show a greater magnitude of effect at genomic sites located near already affected sites.

### 2.3. Stress Hormone-Driven DNA Methylation Changes Quantitatively Accrue at Open Sea, GR Binding, and Chromatin-Accessible Sites

**Figure 3:**
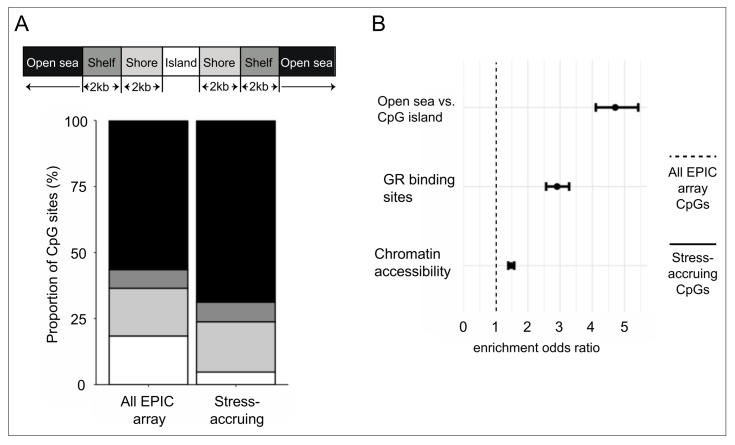
Cortisol-induced DNA methylation changes accrue at open sea, GR binding, and chromatin-accessible sites. (**A**). Stacked bar plot comparing the genomic region annotation of all EPIC array-covered and the stress-accruing CpG sites. (**B**). Enrichment analyses comparing the functional annotation of all EPIC array-covered CpG sites with that of the stress-accruing CpGs. Error bars show odds ratios and 95% confidence intervals, which were calculated with Fisher’s exact test.

Subsequent analyses aimed to better characterize CpG sites with potential cumulative cortisol-induced changes in DNA methylation. Among the 5820 CpG sites undergoing cortisol-induced methylation changes at both middle and late passage (Figure 1B,C), a total of 4646 CpGs exhibited a consistent direction of change (hyper- or hypo-methylation) at both timepoints and greater cortisol-induced changes at late as compared to middle passage, i.e., cumulative cortisol-induced DNA methylation changes. These sites are hereinafter denoted by “stress-accruing CpGs” and described in more detail in Supplementary Table S1. As compared to all EPIC array-covered CpG sites, the stress-accruing CpGs were more likely to be annotated at open sea genomic regions as opposed to CpG islands (OR 4.7, 95% CI 4.1 to 5.4) and to be localized within GR ChIP-seq peaks previously identified in IMR-90 cells [32] (OR 2.9, 95% CI 2.6 to 3.3) and accessible chromatin regions defined by ENCODE-derived DNase hypersensitivity sites (OR 1.5, 95% CI 1.4 to 1.6) (all *p*-values < 2.2 × 10^−16^; Figure 3A,B). Taken together, these findings support local genomic context as an important determinant of cortisol-induced methylation changes, which tend to quantitatively accrue at open sea, GR binding, and chromatin-accessible sites.

## 3. Discussion

Environmental stress has been proposed to impact cell function and phenotypic outcomes via DNA methylation changes [4,6,7,8,9], and prolonged stress exposure has been further hypothesized to induce cumulative methylation changes at susceptible genomic sites [6,11]. Building on this hypothesis, the present study shows that prolonged glucocorticoid stress hormone (cortisol) exposure in cell culture induces profound and widespread methylomic changes that emerge at selected CpG sites, are more likely to spread to nearby located CpGs, and quantitatively accrue at open sea, GR-susceptible, and chromatin-accessible sites.

By assessing multiple timepoints (early, middle, late passage) during prolonged (51-day) exposure to naturalistic cortisol levels, the present study identified a spatiotemporal pattern of cortisol-induced methylomic changes that appear to affect an exponentially increasing number of CpG sites with time and are more likely to emerge near already affected sites. These results extend prior work showing that DNA methylation changes gradually emerge across juxtaposed CpGs during a shorter (4-day) treatment with the synthetic (and much more potent) glucocorticoid dexamethasone [14], while also suggesting that widespread epigenetic changes become established over longer timescales during exposure to naturalistic cortisol levels. Although no studies have compared the methylome-wide effects of cortisol and dexamethasone, differences in the time-course and strength of establishment are plausible given that synthetic glucocorticoids have transcriptional effects markedly different from those of naturally occurring glucocorticoids [33]. Such differences highlight the importance of considering compound type and concentration when modeling stress in cell culture. Taken together, these results also build on prior work to support a “stress-driven epigenomic seeding and spreading” hypothesis, which could be explained by DNA methylation-dependent gating of local chromatin accessibility and transcription factor binding [14,17,19,31]. This intriguing hypothesis remains to be further tested and characterized by studies that include additional timepoints and more comprehensive epigenomic profiling.

Another key observation is that cortisol-induced methylation changes can quantitatively accrue over time at selected CpG sites. Such CpGs could serve as epigenetic signatures and potential biomarkers of prolonged glucocorticoid and stress exposure. Supporting this possibility, the extent of methylation changes at selected CpGs of the glucocorticoid-responsive *FKBP5* gene has been shown to correlate with the total burden of glucocorticoid exposure in mice [34]. Notably, the stress-accruing CpGs show strong enrichment for localization at open sea, GR binding, and chromatin-accessible sites. These localization findings are in line with previous work in both humans and cells. Specifically, CpG sites located at open sea regions are more frequently associated with environmental stress, such as childhood maltreatment and natural disasters [35,36], and CpGs co-localizing with GR binding sites are more likely to show methylation changes as a result of both cumulative lifetime stress and dexamethasone administration [16]. Furthermore, the methylation status at individual CpG sites has been shown to correlate with their chromatin accessibility at the single-cell level [37]. Together these findings suggest that local genomic context is an important determinant of the methylomic changes induced by cortisol and may thus play crucial roles in shaping the epigenomic effects of stress over time.

The findings of the present study should be viewed within the context of its limitations. The EPIC array interrogates most human genes (~99% of all known genes), yet only sparsely covers the human methylome (~3% of all CpG sites). Thus, it is possible that stress-driven methylation changes at CpG sites not covered by the array could follow principles considerably different from those observed here, and this possibility can only be addressed by more comprehensive profiling methods, such as whole-genome bisulfite sequencing. Moreover, this study characterized the methylomic effects of prolonged cortisol exposure but did not distinguish between effects mediated by physical interaction of the GR with genomic sites and other effects of glucocorticoid signaling. For example, it is possible that many epigenomic effects do not require GR binding to the genome but can occur due to glucocorticoid effects on fundamental cell processes, such as proliferation and metabolism. Cell proliferation is itself associated with methylation changes [38], and future studies may determine the extent to which the present findings generalize to relevant non-replicating cells, such as neurons. Lastly, the present study included a considerable number of biological replicates to increase the power for detecting cortisol-induced methylation changes but only assessed a limited number of time snapshots. Assessing additional timepoints will provide a more nuanced understanding of how stress hormone-driven methylomic changes become established along the cellular lifespan.

In conclusion, the present study shows that prolonged exposure to naturalistic stress hormone levels induces profound and widespread methylomic changes that emerge, spread, and accrue over time in a site-dependent manner. Follow-up cell culture and in vivo studies that employ more comprehensive epigenomic profiling, additional timepoints of assessment, and targeted experimental manipulations may provide deeper insights into how prolonged stress impacts the epigenome to shape phenotypic outcomes.

## 4. Materials and Methods

### 4.1. Cell Culture and Treatments

Human IMR-90 (female fetal lung fibroblast) cells were obtained from the Coriell Institute Cell Repository and were maintained in no-phenol-red Dulbecco’s Modified Eagle Medium (DMEM) supplemented with 15% fetal bovine serum, high glucose, sodium pyruvate, l-glutamine, non-essential amino acids, and antibiotic/antimycotic. Cells were grown in a humidified incubator at 37 °C and 5% CO_2_. All cultures were seeded at a cell density of 10,000 cells/cm^2^ and allowed to proliferate for four to five days or until the cells reached 90% confluency. Cortisol was diluted in a very low final amount of DMSO (0.0001%) and continuously added to cultures for the indicated duration of time at a final concentration of 100 nM. The same final DMSO concentration was used as a vehicle control. Both compounds were purchased from Sigma-Aldrich (St. Louis, MO, USA) and were replaced in fresh media simultaneously for both groups every two to three days throughout treatment. IMR-90 cells were then collected while still replicating at early, middle, and late passage after receiving 0, 24, or 51 days, respectively, of continuous treatment with cortisol or vehicle for downstream DNA extraction and methylation measurements. Each treatment condition included six biological replicates.

### 4.2. DNA Methylation Measurements

DNA was extracted using the Genfind V3 DNA extraction kit (Beckman Coulter, Brea, CA, USA) according to manufacturer instructions. DNA concentration and purity were determined using the Take3 microplate spectrophotometer (BioTek Instruments, Winooski, VT, USA). Extracted DNA was bisulfite converted using EZ-96 DNA Methylation Kit (Zymo Research, Irvine, CA, USA), and genome-wide DNA methylation was measured with the Illumina Infinium HumanMethylationEPIC BeadChip (EPIC), which assays DNA methylation at >850,000 CpG sites [39]. According to standard quality control procedures, the following probes were removed: (i) previously identified cross-reactive and polymorphic probes [40]; (ii) CpG probes with less than 3 beads or with a detection *p*-value over 1% in at least 1% of the samples assayed; (iii) CpG probes mapping to the Y chromosome (given that IMR-90 cells originate from a female fetus); and (iv) probes containing SNPs with a minor allele frequency >1% located within 10 bases of the CpG. Following this standardized procedure, a total of 709,065 CpG was included in subsequent analyses. Raw signal intensities were normalized with subset quantile normalization available in the minfi package [41]. Normalized intensity values were then converted into beta values, which were used in all analyses. To adjust for technical batch effects, EPIC arrays were run after randomizing DNA samples from different cell passages and treatment conditions across plate, chip, row, and column. The lack of significant batch effects in the data was confirmed with iterative covariate inspection after principle component analysis, as implemented in the RaMWAS pipeline [42,43].

### 4.3. Statistical Analyses

To identify CpG sites undergoing methylation changes during prolonged cortisol exposure, linear regression models tested the effect of the interaction between treatment condition (cortisol vs. vehicle) and duration (early, middle, and late passage) on methylation levels at each EPIC array-covered CpG. Student’s *t*-test assessed methylation differences between cortisol and vehicle groups at middle and late passage. The Spearman method was used to test the correlation between the cortisol-induced methylation change at each spreading CpG and the distance from the closest seeding CpG. To assess the localization of CpGs within GR binding sites, analyses used online available GR ChIP-seq peaks previously identified in IMR-90 cells [32]. Localization for genomic region (open sea, shelf, shore, CpG island) and ENCODE-derived DNase hypersensitivity sites was performed using the EPIC array annotation (manifest file) derived through the minfi package [41]. Enrichment analyses were performed using Fisher’s exact test with the indicated EPIC array CpGs as background. All *p*-values were 2-tailed, correction for multiple testing was done with the false discovery rate method, and results were considered statistically significant based on an adjusted threshold of α = 0.05. All statistical analyses were conducted in R version 3.6.3.

## Data Availability

Not applicable.

## Supplementary Materials

Supplementary materials can be found at https://www.mdpi.com/article/10.3390/ijms22168778/s1.
